# Glances at the Hospitals

**Published:** 1900-09-01

**Authors:** 


### GLANCES AT THE HOSPITALS.

### THE NORFOLK AND NORWICH HOSPITAL.

THE total amount received during the year was £8,057, and the total expenditure was £9,974, and it is pointed out that the gross income was £2,122 less than in 1898. Special appeals have been made, with the result that an additional £800 have been received, but still the income is considerably under the expenditure, and it is clear that if the former cannot be increased the latter must be diminished by closing part of the Infirmary, a proceeding which we are sure would be most repugnant to the feelings of the Governors. In drawing attention to this deficiency in ordinary income, it ought also to be widely known that Mr. Cadge has presented a second gift of £10,000, and that the Earl of Leicester has given £15,000. The latter sum will not only meet the building expenses of the new Nurses' Home, but will permit of a considerable sum being invested. On the last day of 1898, the hospital contained 140 patients, and 1,532 were admitted during 1899, making a total of 1,672. Of these 505 were discharged recovered, 420 relieved, 349 were made out-patients or sent to convalescent home at Cromer, 34 were discharged for special reasons, 98 died, and 126 remained in the hospital. Of the deaths, 22 took place within 24 hours of admission, and 22 within one week. The coroner held inquests in 12 cases. The out-patients reached the number of 6,158. The analytical table is very carefully compiled, and gives the ages of the patients and the results; but when we turn to the table of operations we find no results shown, and it is necessary to turn back to the analytical table. Even then it is not always possible to pick out the desired information. For instance, we see that seven operations were performed for the radical cure of hernia, and ten for strangulated hernia. Naturally, we should like to know the results, and we refer to the analytical table, where we find that 32 cases of hernia were under treatment, and that 19 of these were cured, four were relieved, three were made out-patients, five died, and one remained under treatment; but we have still to learn the mortality among the seven operations for the radical cure. Again, 559 operations were performed during the year, and it would be most interesting to know which anæsthetics were preferred. Many hospitals now publish such a table.

### ROYAL SEA-BATHING HOSPITAL AT MARGATE.

THIS hospital was founded as far back as 1791, and it must have witnessed vast changes during the 110 years of its existence. On the 1st of January, 1899, it contained 60 patients. 512 cases were admitted during the year and 487 were discharged. Of the latter 59 were cured; 233 greatly benefited; 147 benefited; 42 not improved, and 6 died. The vast majority of the cases treated were afflicted with tuberculous diseases in one of its many forms. There were 253 operations performed during the year, and of these 53 were for excision of diseased glands. It is stated that the principles relied on in the treatment of tuberculous joint diseases have been rest, combined with good feeding and fresh air, rather than operation, "and without exception, early cases have done exceedingly well"; but the broad lines of treatment are classified as follows:—1st, Liberal diet, fresh air and tonics; 2nd, Sea-bathing; 3rd, Surgery. We are pleased to note that the hospital was the recipient during 1899 of two handsome legacies, one of £10,000 and one of £5,000. The board were compelled to use part of this money for current expenses, "as the demands had been so great in regard to the renewal of the hospital and other expenses in connection therewith." Still the general income has increased, but so have the expenses, and everyone connected with infirmaries knows how difficult it is to prevent this, as modern methods and modern appliances must be adopted if the institution is to kept abreast of the times. The average cost per bed occupied was almost 27s.

